# A Quality Improvement (QI) Project to Improve Adherence to Alcohol Dependence Guidelines in an Acute Adult Inpatient Setting

**DOI:** 10.1192/bjo.2026.11504

**Published:** 2026-06-30

**Authors:** Atakan Tum, Suranjana Senanayake, Alkim Arikan, Deepa Bagepalli Krishnan, Michael Rajendram

**Affiliations:** 1 Nottinghamshire Healthcare NHS Foundation Trust, Nottingham, United Kingdom; 2 University of Nottingham, Nottingham, United Kingdom

## Abstract

**Aims:**

The Alcohol Dependence Guidelines provide the standard for assessment and management of alcohol withdrawal for inpatient units in Nottinghamshire Healthcare NHS Foundation Trust. Compliance with this guideline on the Cedar ward has been inconsistent. This could potentially impact patient safety. Using a problem-focused, collaborative approach, this project aimed to initially measure baseline compliance, identify barriers, and implement changes to improve compliance with the guideline by the end of March 2026.

**Methods:**

A retrospective analysis of admissions from August 2025 to March 2026 was used to establish baseline compliance across the various guideline domains (documentation, monitoring, prescribing, and clinical interventions). Data has been collected prospectively to evaluate the impact of implemented change ideas. Multidisciplinary team meetings, survey questionnaires and one to one staff conversations were used to identify barriers for guideline compliance. Changeideas collaboratively identified using a driver diagram included structured teaching sessions for nursing staff and resident doctors, incorporation of alcohol-withdrawal guidance into nursing induction, electronic prescribing notifications prompting CIWA-Ar completion, integration of CIWA-Ar and SADQ tools into the Trust electronic record, and liaison with the Nottinghamshire Area Prescribing Committee to review andupdate existing guidance. Agreed changes were introduced using Plan–Do–Study–Act(PDSA) cycles, with ongoing communication with clinical teams.

**Results:**

Baseline audit findings showed low compliance with several core components of the guideline. Weekly alcohol intake (SADQ score) was documented in 44.4% of cases. Regular chlordiazepoxide prescribing in accordance with guidelines was noted in 55.6% cases, with much lower compliance for as required chlordiazepoxide prescriptions. Escalation of appropriate concerns for clinical review was documented in only 25% of cases. CIWA-Ar was completed in 23.97% of cases. A questionnaire survey of resident doctors identified gaps in awareness and challenges related to processes on the inpatient ward. This suggested that both knowledge gaps and systemic barriers contributed to the problem.

**Conclusion:**

The initial audit confirmed poor compliance with alcohol withdrawal guidelines on Cedar ward, with gaps in documentation, clinical knowledge and awareness identified as key root causes. Implemented changes, such as multidisciplinary teaching on the ward, inclusion of teaching in nursing induction, and prompts in electronic records have improved clinician confidence, recognition, and documentation of withdrawal symptoms. Several process changes being implemented including updating the current guidelines. This is expected to address the root causes of poor compliance with a potential for wider Trust-wide adoption.

